# Factors associated with low patient satisfaction in out-of-hours primary care in Denmark - a population-based cross-sectional study

**DOI:** 10.1186/s12875-017-0681-6

**Published:** 2018-01-11

**Authors:** Mette Tranberg, Peter Vedsted, Bodil Hammer Bech, Morten Bondo Christensen, Søren Birkeland, Grete Moth

**Affiliations:** 1 Department of Public Health Programmes, Randers Regional Hospital, Østervangsvej 68, 8930 Randers NØ, Denmark; 2 Research Unit for General Practice, Department of Public Health, Aarhus University, Bartholins Allé 2, 8000 Aarhus C, Denmark; 3 Section for Epidemiology, Department of Public Health, Aarhus University, Bartholins Allé 2, 8000 Aarhus C, Denmark; 4 Department of Psychology, Faculty of Health Sciences, University of Southern Denmark, Campusvej 55, 5230 Odense M, Denmark

**Keywords:** Out-of-hours care, General practice, Denmark, Patient satisfaction, Delivery of health care

## Abstract

**Background:**

Low patient satisfaction with the quality of out-of-hours primary care (OOH-PC) has been linked with several individual and organizational factors. However, findings have been ambiguous and may not apply to the Danish out-of-hours (OOH) setting in which general practitioners (GPs) perform the initial telephone triage. This study aimed to identify patient-related, GP-related and organizational factors associated with low patient satisfaction.

**Methods:**

The study was based on data from a 1-year population-based survey of OOH-PC (LV-KOS) in the Central Denmark Region in 2010–2011. GPs on OOH duty completed an electronic questionnaire in the OOH computer system, and the registered patients received a subsequent postal questionnaire focusing on contact evaluation, waiting time, demographic characteristics and general self-perceived health. Associations were analysed using multivariable logistic regression with dissatisfaction as the dependent variable.

**Results:**

The patient response rate was 50.6%. For all contact types, 82.5% of the patients were satisfied with the OOH-PC service. More patients were dissatisfied with telephone consultations than with clinic consultations or home visits (8.5% vs. 6.0% and 4.3%, respectively). Contacts assessed by the GP as ‘not severe’ were associated with dissatisfaction for telephone consultations and home visits. Poor general self-perceived health was associated with dissatisfaction for all contact types. Living in urban areas was associated with dissatisfaction for telephone consultations, while unacceptable waiting time was associated with dissatisfaction for all contact types.

**Conclusions:**

We found a high level of patient satisfaction with the OOH-PC service. The only factors affecting patient satisfaction across all contact types were unacceptable waiting time and poor general self-perceived health. For the other investigated factors, patient satisfaction depended on the type of contact. Generally, patients contacting for GP-assessed non-severe health problem and patients living in urban areas were more dissatisfied.

**Electronic supplementary material:**

The online version of this article (10.1186/s12875-017-0681-6) contains supplementary material, which is available to authorized users.

## Background

General practitioners (GPs) form the first line in the Danish healthcare system and are gatekeepers to specialist care. All citizens have free access to medical advice and health care [1, 2], including out-of-hours (OOH) services, which is run by GPs on a rota basis. In four out of the five existing region-based out-of-hours primary care (OOH-PC) cooperatives, GPs are responsible for providing health care for the citizens (0.6–1.8 million inhabitants) from 4 p.m. to 8 a.m. on weekdays and throughout weekends and public holidays [1, 3]. Unlike the OOH-PC settings in many other countries [3], all patient calls are answered and triaged by GPs [4]. They provide advice, write prescriptions, order home visits or refer to clinic consultations or directly to hospital. Most calls (59%) are terminated as telephone consultations [4].

The patient-perceived quality is a crucial element when assessing the provided OOH health care as identified factors associated with low patient evaluation may provide useful information in a quality improvement context [5, 6]. Previous studies of patient satisfaction in the United Kingdom [7–10], the Netherlands [11, 12] and Denmark [1, 13, 14] show that patients are generally satisfied with the OOH-PC service. Yet, the degree of satisfaction is lower for patients who receive telephone consultations than for patients who receive clinic consultations or home visits [1, 9, 11, 13, 15, 16].

Patient-related factors such as socio-economic status [17, 18], age [4, 8–10, 18], gender [4, 9], chronic disease [6] and self-perceived health [17, 19] are related to patient satisfaction. The same is the case for GP-related factors (e.g. age, gender and GP-assessed severity of the health problem) [4, 20, 21] and organizational factors (e.g. waiting time before consultation) [9–11, 16]. However, many of these findings are ambiguous [7, 8, 10, 17, 18] and may not be representative in a Danish OOH-PC setting.

We aimed to identify patient-related, GP-related and organizational factors associated with low patient satisfaction with telephone consultations, clinic consultations and home visits in the Danish OOH-PC service.

## Methods

### Design and setting

This cross-sectional study was based on data from a 1-year population-based survey of OOH-PC, *‘Kontakt-og sygdomsmønster i lægevagten (LV-KOS)*, which was conducted on 1 June 2010–31 May 2011 in the Central Denmark Region, which is the largest of the four Danish region with a primary care-based OOH service (approximately 1.3 million citizens) [22]. The LV-KOS study has been described in detail elsewhere [22]

### Data collection

A “pop-up” questionnaire, which was integrated into the existing electronic patient record system, appeared for the participating GPs after termination of every 10th telephone contact, every 3rd clinic consultation and every home visit. This sampling method was chosen to ensure sufficient numbers of registered clinic consultations and home visits to make valid estimates and yet avoid a considerable increase in the GP workload [22]. The questionnaire covered a range of items, e.g. about the patient’s reasons for contacting and the GP-assessed severity of the presented health problem. Two to five days after the OOH-PC contact, the GP-registered patients received a postal questionnaire about their experience with the OOH-PC service, including general self-perceived health and general satisfaction with the contact. Only patients older than 18 years were asked about their general self-perceived health. Questionnaires regarding contact with children below 18 years of age were sent to the parents [22]. A reminder was sent in case of no response after two weeks [22]. Details of the GP and patient questionnaires are reported elsewhere [22].

### Study population

In total, 18,267 patient contacts (telephone consultations, clinic consultations and home visits) were systematically registered by GPs. A total of 4015 (22%) were excluded from the patient survey, and these patients received no questionnaire. Reasons for exclusion were: protection against research participation (*n* = 2417 (60.2%)), previous inclusion of same patient (*n* = 863 (21.5%)), unknown postal address (*n* = 102 (2.5%)), sensitive matters (e.g. attempted suicide or terminal illness) (*n* = 377 (9.4%)) and death (*n* = 256 (6.4%)).

### Data management

We included the following patient-related factors: patient age and gender, general self-perceived health, chronic disease, residence and employment status. We defined GP age, GP gender and GP-assessed severity of the health problem as GP-related factors. Finally, we included the patient-perceived waiting time before the received service as an organisation-related factor. The electronic OOH-PC database provided data on date and type of contact, GP age and gender, patient age and gender, and patient name and postal address [22]. Patient age was categorized into five groups: 0–4 years, 5–18 years, 19–50 years, 51–75 years and >75 years. This categorization was chosen based on the pattern of disease and the density of contacts to the OOH-PC service; patients aged 0–4 years were considered a special group and was analysed separately. The GP questionnaire also included information on GP-assessed severity of the health problem; the original five response categories were: “Severe”, "Potentially severe and the patient needs to be seen", “Not severe”, “Not ill” and “Don’t know”. When used for logistic regression analyses, this variable was dichotomised into “Potentially severe” (combining “Severe” and “Potentially severe and the patient needs to be seen”) and “Not severe” (combining “Not severe” and “Not ill”). The answer “Don’t know” was not included in the analyses (*n* = 2). We included a question on general self-perceived health inspired by the Short Form-12 (SF-12) questionnaire [23]. The five options for answers were: “Excellent”, “Very good”, “Good”, “Less good” and “Poor”. In the logistic regression analyses, these were dichotomised into “Good” (combining “Excellent”, “Very good” and “Good”) and “Poor” (combining “Less good” and “Poor”) when included in the logistic regression analysis, as done in another study [24]. The patients were asked whether they had chronic disease(s) and whether they found the waiting time (before their consultation) acceptable. The original four answers were: “Yes”, “No”, “Neutral” and “Don’t know”. In the logistic regression analyses, the latter variable was dichotomised into “Acceptable waiting time” (“Yes”) and “Unacceptable waiting time” (“No”). The answers “Neutral” and “Don’t know” were not included in the analyses because answering “Neutral” or “Don’t know” is not comparable with experiencing acceptable or unacceptable waiting time. In total, 93 and 870 patients answered “Don’t know” or “Neutral”, respectively, corresponding to 1.3% and 12.1% of the study population. Supplementary analyses showed that findings largely remained unchanged when including, for example, neutral respondents in the acceptable waiting group (data not shown).

The overall satisfaction with the contact was rated by the patient through selection of one of six response options: “Very satisfied”, “Satisfied”, “Dissatisfied”, “Very dissatisfied”, “Neutral” and “Don’t know”. These were dichotomised into “Satisfied” (combining “Very satisfied” and “Satisfied”) and “Dissatisfied” (combining “Dissatisfied” and “Very dissatisfied”) when used in the logistic regression analyses. The answers “Neutral” and “Don’t know” were not included in the analyses. The rationale for not including these answers is that answering “Neutral” or “Don’t know” cannot be unambiguously classified in any of the two categories. A total of 655 patients answered “Neutral”, corresponding to 9.1% of the study population. However, supplementary analyses showed that findings largely remained unchanged when including, for example, neutral respondents in the satisfied group (data not shown). Additionally, only a small proportion of patients answered “Don’t know”, corresponding to 0.8% of the study population. English translations of the used questions are presented in Additional file 1.

Patient residence was grouped into “Urban area” (≥34,000 inhabitants) and “Rural area” (<34,000 inhabitants) based on the patient’s postal code. Patients living outside the Central Denmark Region were scored as “unknown”. Distance to emergency departments may affect the patient’s consumption of OOH-PC services. As the distance is longer in rural areas and shorter in urban areas, we considered population density to be the most appropriate basis for classification of the patients’ residence. Patients living outside the Central Denmark Region were excluded (e.g. tourists) because our evaluation focused on the experience of the OOH-PC service among ordinary patients.

The Danish Register for Evaluation of Marginalization (DREAM) provided information on the patient’s employment status [25]. This variable was used as a proxy measurement for socio-economic status [25]. Based on the DREAM code for the week of the OOH-PC contact, employment status was categorized as “Self-supporting” (coded with receipt of no benefit in DREAM; maternity leave or student), “Pensioners and retirees” (coded with receipt of benefits related to old age or early retirement) and “Not self-supporting” (coded with receipt of partial or full disability pension, social assistance or sick leave)*.*

### Statistical analyses

The chi-square test was used to test differences between respondents and non-respondents in terms of age, gender and residence. Descriptive statistics were used to summarize patient characteristics for each type of contact. Logistic regression was used to estimate crude and adjusted odds ratios (ORs) for overall dissatisfaction with the OOH-PC service in terms of patient-related, GP-related and organizational factors. Unadjusted analyses were performed for each independent variable. These analyses were followed by analyses adjusted for statistically significant variables (*p* < 0.01) from the univariate analyses. This led us to include residence, general self-perceived health, self-reported chronic disease, employment status, GP age, GP-assessed severity of contact and patient-perceived waiting time before contact in the adjusted analyses. We also included patient age [4, 8–10, 18], patient gender [4, 9] and GP gender [20, 21] in the adjusted analyses because they were found to be important confounding variables in the literature.

Patient age was entered as a continuous variable. The small number of dissatisfied patients made it impossible for us to include all variables in the models. Therefore, we made different models, i.e. included different numbers of confounding variables in the analyses, because we had to prioritise and include only the most important confounding variables in our models to avoid getting an unstable model with wide confidence intervals. The selection of confounding variables in the models was based on excluding any variable that did not fulfil the criteria to be considered as a valid confounder in the exact model (i.e. not associated with the exposure) and thus was expected to have little effect on the OR estimate. To evaluate the robustness of our assumptions, the excluded variables were initially included in the models. If including the variable did not affect the results, this variable was not retained in the adjusted model. In total, four different adjusted models were used to measure the associations between the patient-related factors and dissatisfaction. Model 1: Associations between the patient’s residence and dissatisfaction included patient’s gender, age, general self-perceived health, self-reported chronic disease, employment status and GP-assessed severity of contact as confounding variables. The variable patient-perceived waiting time before contact was excluded. Model 2: Associations between the patient’s general self-perceived health and dissatisfaction included patient’s gender, age, residence, self-reported chronic disease, patient-perceived waiting time before contact and GP-assessed severity of contact as confounding variables. The variable employment status was excluded. Model 3: Associations between self-reported chronic disease and dissatisfaction included patient’s gender, age, residence, general self-perceived health and GP-assessed severity of contact as confounding variables. The variables employment status and patient-perceived waiting time before contact were excluded. Model 4: Associations between the patient’s employment status and dissatisfaction included patient’s gender, age, residence, general self-perceived health, self-reported chronic disease and GP-assessed severity of contact as confounding variables. The variable patient-perceived waiting time before contact was excluded.

Three adjusted models were used to measure associations between GP and organizational-related factors and dissatisfaction. Model 1: Associations between GP gender and dissatisfaction and between GP age and dissatisfaction included the same confounding variables. These were patient’s gender, age, general self-perceived health, patient-perceived waiting time before contact and GP-assessed severity of contact as confounding variables. The variables GP age, patient residence, self-reported chronic disease and employment status were excluded. Model 2: Associations between GP-assessed severity of contact and dissatisfaction included patient’s gender, age, residence, general self-perceived health, self-reported chronic disease, patient-perceived waiting time before contact, GP age and gender as confounding variables. Employment status was excluded from the model. Model 3: Associations between patient-perceived waiting time before contact and dissatisfaction included patient’s gender, age, residence, general self-perceived health, self-reported chronic disease and employment status as confounding variables. The variable GP-assessed severity of contact was excluded from the model.

As different patients may have been in contact with the same GP, we accounted for GP clustering by using robust standard errors in all unadjusted and adjusted models [26]. Due to the sampling method (one in ten telephone contacts, one in three clinical consultations and every home visit), all statistical analyses were performed separately for each contact type. *P*-values of 0.05 or less were considered statistically significant. All statistical analyses were conducted using Stata 11.0. (StataCorp LP, College Station, TX, USA).

### Ethical approvals

The study was approved by the Danish Data Protection Agency (j.no. 2013–41-1594). Approval from the Danish National Committee on Health Research Ethics was not required as no biomedical intervention was performed in this study. According to Danish law, confidentiality aspects and associated bioethical issues in non-interventional health care research are managed by the Data Protection Agency and governed by the Danish Act on Processing of Personal Data. Informed consent from participants and GPs was obtained through their acceptance to fill out the questionnaires.

## Results

In total, 14,252 (75.1%) patients received a postal questionnaire, and 7213 patients responded (50.6%). No statistically significant differences were found between respondents and non-respondents for gender, except for home visits for which a higher proportion of men responded (*p* < 0.001) (Table 1). Statistically significantly higher response rates were found for respondents in rural areas. Patients aged 19–50 years comprised the highest proportion of respondents for telephone consultations and clinic consultations, whereas patients aged 51–75 years formed the highest proportion of respondents for home visits.

**Table 1:** Comparison between respondents (*n* = 7213) and non-respondents (*n* = 7039) in terms of gender, age group and residence

	Telephone consultations			Clinic consultations			Home visits		
	Non-respondents *n* = 1899 (100%)	Respondents *n* = 1789 (100%)	*P*-value^a^	Non-respondents *n* = 2521 (100%)	Respondents *n* = 3183 (100%)	*P*-value^a^	Non-respondents *n* = 2619 (100%)	Respondents *n* = 2241 (100%)	*P*-value^a^
	n (%)	n (%)		n (%)	n (%)		n (%)	n (%)	
Gender									
Male	888 (46.8)	825 (46.1)	0.679	1236 (49.0)	1541 (48.4)	0.645	1233 (47.0)	1124 (50.2)	<0.001
Female	1011 (53.2)	964 (53.9)		1285 (51.0)	1642 (51.6)		1386 (53.0)	1117 (49.8)	
Age groups (years)									
0–4	329 (17.3)	482 (26.9)	<0.001	580 (23.0)	931 (29.3)	<0.001	170 (6.5)	200 (8.9)	<0.001
5–18	257 (13.5)	298 (16.7)		439 (17.4)	679 (21.3)		140 (5.3)	155 (6.9)	
19–50	817 (43.0)	568 (31.8)		1180 (46.8)	947 (29.8)		583 (22.3)	405 (18.1)	
51–75	297 (15.6)	337 (18.6)		278 (11.0)	549 (17.3)		726 (27.8)	832 (37.1)	
> 75	199 (10.5)	104 (15.8)		44 (1.7)	77 (2.4)		1000 (38.2)	649 (29.0)	
Patient’s residence									
Urban area	939 (49.4)	824 (46.1)	<0.001	1368 (54.3)	1523 (47.9)	<0.001	1178 (45.0)	871 (38.9)	<0.001
Rural area	893 (47.0)	918 (51.3)		1049 (41.6)	1569 (49.3)		1409 (53.8)	1357 (60.6)	
Missing data	67 (3.5)	47 (2.6)		104 (4.1)	91 (2.9)		32 (1.2)	57 (0.6)	

^a^Tested using a chi-square test

The overall satisfaction and contact characteristics are shown in Table 2. For all contact types, 6.1% of the patients were dissatisfied and 82.5% were satisfied with the OOH-PC service. Patients receiving telephone consultations were more often dissatisfied than patients receiving clinic consultations or home visits (*p* < 0.001). The proportion of patients who found that they had experienced unacceptable waiting time was highest among patients receiving clinic consultations. Patients reporting to find the waiting time unacceptable were mostly women, aged 19–50 years and living in rural areas (data not shown).

**Table 2:** Contact characteristics

	Telephone consultations		Clinic consultations		Home visits		*P*-value^a^
	n	(%)	n	(%)	n	(%)	
Number of patient contacts (*n* = 7213)	1789	(100)	3183	(100)	2241	(100)	
Overall satisfaction							
Satisfied	1379	(77.0)	2656	(83.4)	1916	(85.5)	<0.001
Dissatisfied	152	(8.5)	192	(6.0)	98	(4.3)	
Neutral	214	(12.0)	301	(9.5)	140	(6.3)	
Don’t know	17	(1.0)	11	(0.4)	28	(1.3)	
Missing data	27	(1.5)	23	(0.7)	59	(2.6)	
Organizational factor:							
Patient-perceived waiting time							
Acceptable waiting time	1325	(74.1)	2279	(71.8)	1647	(73.5)	<0.001
Unacceptable waiting time	174	(9.7)	459	(14.4)	258	(11.5)	
Neutral	231	(12.9)	387	(12.2)	252	(11.2)	
Don’t know	31	(1.7)	24	(0.8)	38	(1.7)	
Missing data	28	(1.6)	34	(1.1)	46	(2.1)	
Patient-related factors:							
Age groups (years)							
0–4	482	(26.9)	931	(29.3)	200	(8.9)	<0.001
5–18	298	(16.7)	679	(21.3)	155	(7.0)	
19–50	568	(31.8)	947	(30.0)	405	(18.1)	
51–75	337	(18.8)	549	(17.3)	832	(37.3)	
> 75	104	(5.8)	77	(2.4)	649	(29.0)	
Gender							
Male	825	(46.1)	1541	(48.4)	1124	(50.2)	<0.001
Female	964	(53.8)	1642	(51.6)	1117	(49.8)	
Patient’s residence							
Rural area	918	(51.3)	1569	(49.3)	1357	(60.6)	<0.001
Urban area	824	(46.1)	1523	(47.9)	871	(38.9)	
Unknown^b^	47	(2.6)	91	(2.9)	13	(0.6)	
Employment status							
Self-supporting	1287	(71.9)	2563	(80.5)	1140	(50.9)	<0.001
Pensioners and retirees	171	(9.6)	244	(7.7)	682	(30.4)	
Not self-supporting	247	(13.8)	325	(10.2)	373	(16.6)	
Missing data	84	(4.7)	51	(1.6)	46	(2.1)	
General self-perceived health^c^							
Excellent	112	(10.9)	179	(11.1)	72	(3.8)	<0.001
Very good	300	(29.3)	580	(36.1)	281	(14.8)	
Good	333	(32.5)	521	(32.4)	582	(30.7)	
Poor	186	(18.2)	244	(15.2)	626	(33.0)	
Very poor	66	(6.4)	50	(3.1)	252	(13.3)	
Missing data	28	(2.7)	34	(2.1)	84	(4.4)	
Self-reported chronic disease							
No chronic disease	1292	(72.2)	2491	(78.3)	1049	(46.8)	<0.001
Chronic disease^d^	497	(27.8)	692	(21.7)	1192	(53.2)	
GP-related factors:							
GP-assessed severity of contact							
Potentially severe	236	(13.2)	1209	(38.0)	1161	(51.8)	<0.001
Not severe	1486	(83.0)	1937	(60.9)	1061	(47.3)	
Don’t know	67	(3.8)	37	(1.2)	17	(0.8)	
Missing data	0	(0.0)	0	(0)	2	(0.1)	

^a^Tested using a chi-square test

^b^Patients living outside the Central Denmark Region

^c^Only patients aged >18 years

^d^See Additional file 1

Table 3 depicts associations between patient-related factors and dissatisfaction. Living in urban areas was statistically significantly associated with dissatisfaction among patients receiving a telephone consultation, but this association was not found for other contact types. Poor general self-perceived health was associated with dissatisfaction for all three contact types. No statistically significant association was seen between self-reported chronic disease and dissatisfaction for any contact type in the adjusted models. No statistically significant association was found between employment status and dissatisfaction although ‘not self-supporting’ tended to be associated with dissatisfaction with clinic consultations.

**Table 3:** Likelihood (OR) of dissatisfaction with different contact types according to patient-related factors

	Telephone consultations		Clinic consultations		Home visits	
	Crude OR (95% CI) *n* = 1,321^a^	Adjusted OR (95% CI) n= 698^a^	Crude OR (95% CI) *n* = 1,397^a^	Adjusted OR (95% CI) n = 1,158^a^	Crude OR (95% CI) n = 1,637^a^	Adjusted OR (95% CI) n = 1,394^a^
Residence						
Rural area (ref)	1	1	1	1	1	1
Urban area	**1.58 (1.10–2.27)**	**1.65 (1.09–2.50)** ^**b**^	1.16 (0.87–1.53)	1.09 (0.73–1.64)^b^	1.46 (0.92–2.30)	1.42 (0.81–2.50)^b^
General self-perceived health						
Good health (ref)	1	1	1	1	1	1
Poor health	**3.22 (2.13–4.88)**	**3.35 (1.78–6.31)** ^**c**^	1.35 (0.85–2.14)	**1.76 (1.02–3.04)** ^c^	**2.16 (1.36–3.42)**	**2.45 (1.33–4.50)** ^**c**^
Self-reported chronic disease						
No chronic disease (ref)	1	1	1	1	1	1
Chronic disease^f^	**2.26 (1.67–3.07)**	1.31 (0.81–2.01)^d^	0.88 (0.60–1.29)	0.78 (0.47–1.30)^d^	1.28 (0.85–1.94)	1.35 (0.74–2.46)^d^
Employment status						
Self-supporting (ref)	1	1	1	1	1	1
Pensioners and retirees	1.07 (0.57–1.99)	0.75 (0.34–1.67)^e^	0.51(0.25-1.07)	1.25 (0.47–3.34)^e^	**0.51 (0.29–0.90)**	0.60 (0.29–1.24)^e^
Not self-supporting	**2.27 (1.50–3.44)**	1.02 (0.59–1.76)^e^	1.37 (0.82–2.03)	1.53 (0.93–2.52)^e^	**1.64 (1.08–2.49)**	1.07 (0.57–2.00)^e^

Statistically significant estimates are shown in bold

^a^The lowest amount of observations in the models

^b^Adjusted for patient’s gender, age, general self-perceived health, self-reported chronic disease, employment status and GP-assessed severity of contact. Including patient-perceived waiting time in the model did not affect the results, and the variable was not retained in the adjusted model

^c^Adjusted for patient’s gender, age, residence, self-reported chronic disease, patient-perceived waiting time and GP-assessed severity of contact. Including employment status in the model did not affect the results, and the variable was not retained in the adjusted model

^d^Adjusted for patient’s gender, age, residence, general self-perceived health and GP-assessed severity of contact. Including employment status and patient-perceived waiting time in the model did not affect the results, and the variables were not retained in the adjusted model

^e^Adjusted for patient’s gender, age, residence, general self-perceived health, self-reported chronic disease and GP-assessed severity of contact. Including patient-perceived waiting time in the model did not affect the results, and the variable was not retained in the adjusted model

^f^See Additional file 1

Table 4 shows associations between GP-related and organizational factors and dissatisfaction. GP assessment of patient contact as non-severe was statically significantly associated with dissatisfaction among patients who received a telephone consultation or home visit. Contact with a GP aged >60 years was statically significantly associated with dissatisfaction among patients receiving a telephone consultation or home visit. For all contact types, patient-assessed unacceptable waiting time was associated with dissatisfaction, while no association was found between GP gender and dissatisfaction.

**Table 4:** Likelihood (OR) of dissatisfaction with different contact types according to GP-related and organizational factors

	Telephone consultations		Clinic consultations		Home visits	
	Crude OR (95% CI) n = 1,321^a^	Adjusted OR (95% CI) n= 698^a^	Crude OR (95% CI) *n* = 2,467^a^	Adjusted OR (95% CI) *n* = 1,157^a^	Crude OR (95% CI) n = 1,749^a^	Adjusted OR (95% CI) n = 1,395^a^
GP-related factors:						
GP gender						
Male (ref)	1	1	1	1	1	1
Female	1.23 (0.84–1.82)	1.14 (0.67–1.94)^b^	0.99 (0.67–1.47)	1.18 (0.73–1.92)^b^	0.68 (0.41–1.11)	0.70 (0.41–1.20)^b^
GP age (years)						
< 41 (ref)	1	1	1	1	1	1
41–50	1.51 (0.94–2.43)	1.25 (0.59–2.63)^b^	**2.16 (1.39–3.37)**	1.03 (0.53–2.00)^b^	1.06 (0.58–1.94)	1.00 (0.48–2.05)^b^
51–60	0.99 (0.65–1.51)	1.30 (0.68–2.50)^b^	**2.01 (1.31–3.07)**	1.33 (0.78–2.29)^b^	1.31 (0.78–2.21)	1.16 (0.60–2.27)^b^
> 60	1.52 (1.00–2.31)	**1.96 (1.04–3.68)** ^b^	1.46 (0.78–2.74)	1.12 (0.51–2.45)^b^	**2.26 (1.03–4.98)**	**2.82 (1.24–6.40)** ^b^
GP-assessed severity of contact						
Potentially severe (ref)	1	1	1	1	1	1
Not severe	**2.41 (1.27–4.58)**	**7.15 (2.49–20.6)** ^**c**^	1.47 (1.00–2.10)	1.28 (0.79–2.09)^c^	**2.69 (1.72–4.02)**	**2.41 (1.38–4.21)** ^**c**^
Organizational factor:						
Patient-perceived waiting time						
Acceptable waiting time (ref)	1	1	1	1	1	1
Unacceptable waiting time	**3.28 (2.00–5.35)**	**3.25 (1.85–5.72)** ^**d**^	**2.75 (1.98–3.83)**	**3.21 (2.03–5.06)** ^**d**^	**4.08 (2.47–6.74)**	**3.83 (2.11–6.96)** ^**d**^

Statistically significant estimates are shown in bold

^a^The lowest amount of observations in the models

^b^Adjusted for patient’s gender, age, general self-perceived health, patient-perceived waiting time, GP-assessed severity of contact. Including GP age, patient residence, self-reported chronic disease and employment status in the model did not affect the results, and the variables were not retained in the adjusted model

^c^Adjusted for patient’s gender, age, residence, general self-perceived health, self-reported chronic disease, patient-perceived waiting time, GP age and gender. Including employment status in the model did not affect the results, and the variable was not retained in the adjusted model

^d^Adjusted for patient’s gender, age, residence, general self-perceived health, self-reported chronic disease and employment status. Including GP-assessed severity of contact in the model did not affect the results, and the variable was not retained in the adjusted model

## Discussion

### Main findings

Overall, 6.1% of patients evaluated the OOH-PC encounter as dissatisfying, while 82.5% of patients were satisfied. Patients receiving a telephone consultation were significantly more dissatisfied than patients receiving a clinic consultation or home visit. Poor general self-perceived health was associated with dissatisfaction for all contact types, while GP-assessed non-severe contacts were associated with dissatisfaction among patients receiving a telephone consultation or home visit. However, having a chronic disease was not in itself associated with dissatisfaction. Patient-assessed unacceptable waiting time was associated with dissatisfaction for all types of contact. Higher GP age was associated with dissatisfaction with telephone consultations and home visits. These findings indicate that patients who perceive their general health as low and are triaged as non-severe contact or triaged by older GPs form a specific group of patients who tend to give negative evaluations of the OOH-PC service. Still, the strongest and most decisive factor for dissatisfaction was unacceptable waiting time.

### Strengths and weaknesses

The study was based on a large sample of contacts to the OOH-PC service, and the large data set ensured high statistical precision. Furthermore, the participating GPs and the randomly included contacts have been demonstrated to be highly representative of all contacts in the OOH-PC service [22]. The core items of the questionnaires had low levels of missing data; this indicates that the relevance of the questions was accepted by the respondents.

The short time period between the contact with the OOH-PC service and the completion of the questionnaire reduced the risk of recall bias. Misclassification of the independent self-reported patient-related and organization-related factors is unlikely to depend on the level of dissatisfaction, so misclassification is not considered to have caused bias in our findings.

Likewise, the random recruitment of patients through electronic “pop-up” questionnaires combined with very high participation rates among GPs also limited selection bias [22]. The overall patient response rate is considered acceptable for a survey in a heterogeneous population [27, 28] and resembles the response rates for other surveys in this field [8, 11, 12, 17, 19]. However, non-respondents differed from respondents in terms of age and residence, whereas no gender differences were found, except for home visits. The fairly low response rate among parents of children aged 5–18 years is difficult to explain, but may be due to reluctance to complete time-consuming paper questionnaires. This group tended to be younger and in better health. They may, therefore, find the questionnaire less relevant than older patients. We found lower response rates among older patients (> 75 years). This finding is consistent with findings made in other studies. The lower response rate in the group of older patients is not surprising as they are generally less resourceful and more ill than younger patients [27]. However, we see no reason why the lower response rates among these groups should be associated with dissatisfaction with their contact to the OOH-PC service; selection bias is thus unlikely to explain our results. This argument is supported by a systematic review of the literature on patient satisfaction, which shows that older respondents and healthier respondents generally report higher levels of satisfaction [29].

If the excluded patients (22%) were more dissatisfied than the included patients, we cannot exclude selection bias. However, the choice to exclude specific patients was based on ethical concerns and logistical issues, e.g. unknown address, and is thus unrelated to the patient’s experience of the contact. Therefore, this exclusion should not have influenced the reported ORs.

Other studies have reported that dissatisfied patients are less likely to participate than satisfied patients [30], and the level of dissatisfaction in our study may thus be underestimated. Further, residual confounding cannot be excluded as some factors, e.g. education level, marital status and ethnicity, have been shown to be related to dissatisfaction in previous studies [9, 19]. Using the OR as a measure may overestimate associations if the prevalence of the outcome measure is high [31]. However, the level of overestimation in our study is considered negligible due to a low prevalence of the outcome measure (less than 7% of the patients were dissatisfied).

As the organization of OOH-PC in Denmark is fairly homogenous, the results of the present study can be generalized to other regions in Denmark [1, 3]. Despite differences between the OOH-PC services provided in Denmark and in other countries, the patient-related findings are likely to be generalizable to other countries with comparable OOH-PC settings that include telephone triage by health professionals.

### Comparison with other studies

Telephone consultations were more often associated with dissatisfaction than clinic consultations and home visits. This finding is in accordance with previous studies conducted in countries with other ways of organizing the OOH-PC service [1, 9, 11, 12, 15]. We do not know the reason for this, but the result is most likely related to variances in patient populations and GP triage [17]. The variance in dissatisfaction scores for different types of contacts should be further investigated in future studies. Our study found that poor general self-perceived health, but not chronic disease, was related to dissatisfaction, which is in line with the findings in other studies [6, 17, 19]. It has been suggested that patients with poor general self-perceived health might be more vulnerable to the physician’s attitude and behaviour [17, 32], and this is likely to be associated with expectations. Previous studies have suggested that a mismatch between the patient’s expectations and the service received has a major impact on the level of satisfaction with the OOH-PC service [7, 16, 18]. This may explain the association found between GP-assessed non-severity of the contact and high level of patient dissatisfaction. Thus, some patients may be particularly dissatisfied with telephone consultations if they expected that they would be offered more comprehensive treatment (clinic consultation or home visit) and/or that the triage GP would rate their health problem as more severe.

Residence in urban areas was statistically significantly associated with more dissatisfaction with telephone consultations. The observed association may, to some extent, be explained by a lower threshold for contacting the OOH-PC service in urban areas, which can lead to a mismatch between expected and received service [7]. Alternatively, this finding might reflect differences in access to general practice as suggested by Wilson et al. [18]. However, the present study is unable to investigate this further. Our finding that higher GP age was associated with dissatisfaction with telephone consultations and home visits is in line with the findings of a previous study, which shows that patients tend to rate younger GPs more positively than older GPs [20, 21]. This might also reflect that older GPs are more experienced and thus may provide a different type of telephone consultation (e.g. shorter consultations and fewer referrals to face-to-face contacts).

In consistence with previous findings, unacceptable waiting time was strongly associated with increased dissatisfaction [9–11, 16]. Measuring the actual (rather than the perceived) waiting time might add relevant information as to whether it would be realistic to address this patient-perceived problem. Patients who found the waiting time unacceptable were often women aged 19–50 years and living in rural areas (data not shown).

In contrast to findings in previous studies, our results showed no association between employment status (i.e. socio-economic status) and dissatisfaction [17, 18]. A possible explanation for this finding may be different categorizations of socio-economic factors in different studies.

## Conclusions and implications

In general, a high level of patient satisfaction with the OOH-PC service was observed. However, we found lower patient satisfaction with the OOH-PC service among patients who received a telephone consultation. Across all contact types, unacceptable waiting time (organizational factor) and poor general self-perceived health (patient factor) were the only factors associated with dissatisfaction. For the other investigated factors, patient satisfaction depended on the type of contact. Generally, patients living in urban areas and patients with GP-assessed non-severe contact were more dissatisfied. Our findings suggest that a multifaceted approach is required if we intend to increase the satisfaction in all patient groups. GPs could be more aware of the expectations and needs in some patient groups. Patients who contacted the OOH-PC with health problems that were assessed by the triage GP as less appropriate for this setting were more often less satisfied with the service provided; this indicates a mismatch between patient expectations and the actual purpose of OOH-PC. Initiatives that may help adjust the patient-perceived waiting times could be an expedient strategy for achieving higher patient satisfaction.

## Additional file

Additional file 1: English translations of the used questions. (DOCX 17 kb)

## Abbreviations

DREAM: The Danish Register for Evaluation of Marginalization
GP: General Practitioners
LV KOS: In Danish: *Kontakt-og sygdomsmønster i lægevagten*
OOH: Out-Of-Hours
OOH-PC: Out-of-hours primary care
