# Supplementary material for: Factors associated with low patient satisfaction in out-of-hours primary care in Denmark - a population-based cross-sectional study
Source: BMC Fam Pract. 2018 Jan 11;19:15. doi: 10.1186/s12875-017-0681-6 (PMC5765708; doi:10.1186/s12875-017-0681-6)
Supplement: Additional file 1: — English translations of the used questions. (DOCX 17 kb) [file 12875_2017_681_MOESM1_ESM.docx]

Additional file 1

**GP-assessed severity of the contact**

The GP-assessed severity of the patient contact was illustrated by the question: How do you assess the patient contact severity medically? Five response options were available: 1. "Severe", 2 ."Potentially severe and the patient needs to be seen" , 3. "Not severe'' , 4."Not ill " and 5. "Don´t know".

**General self-perceived health**

The question about the patient’s self-perceived health (in general) was phrased as follows: How do you rate your overall health? Five response options were available: 1."Excellent", 2. "Very good", 3."Good", 4."Less good" and 5."Poor".

Self-reported chronic disease

The question about the patient’s self-reported chronic disease was worded as follows: Have you, over the past three months, had one or more of the health problems/diseases listed below? Eleven options were listed: 1. "Cardiovascular disease", 2. "Cancer", 3. "Depression", 4. "Mental health problems, incl. anxiety", 5. "Asthma", 6. "Diabetes", 7. "Emphysema (COPD)", 8. "Osteoarthritis", 9. "Osteoporosis", 10. "Other chronic diseases" and 11. "No".

Perceived waiting time

The question about patient-perceived waiting time was worded as follows: Did you find the waiting time before your consultation (telephone/clinic consultation or home visit) acceptable? Four response options were available: 1."Yes", 2."No2, 3."Neutral" and 4. "Don’t know".

Overall satisfaction

The question about overall patient satisfaction was worded as follows: How satisfied are you overall with the consultation that you received? Six response options were available: 1."Very satisfied", 2."Satisfied" , 3."Dissatisfied", 4. "Very dissatisfied", 5. "Neutral" and 6."Don´t know".
